# Supplementary material for: Lipophilic Grape Seed Proanthocyanidin Exerts Anti-Cervical Cancer Effects in HeLa Cells and a HeLa-Derived Xenograft Zebrafish Model
Source: Antioxidants (Basel). 2022 Feb 19;11(2):422. doi: 10.3390/antiox11020422 (PMC8869705; doi:10.3390/antiox11020422)
Supplement: Supplementary file 1 [file antioxidants-11-00422-s001.zip › antioxidants-1587558-supplementary.pdf]

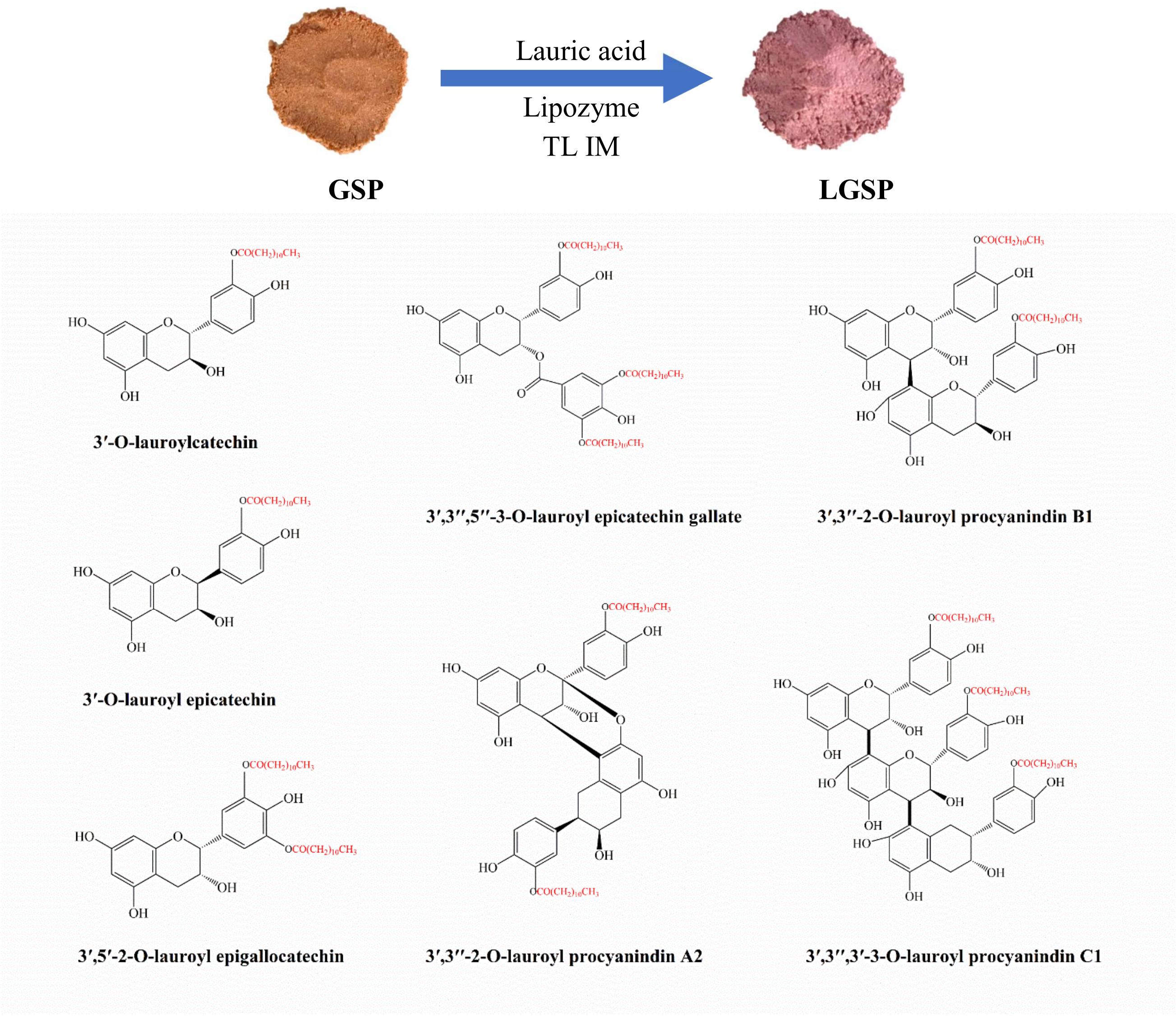

Figure S1. Synthesis of LGSP.
